# Diagnosis of *Coxiella burnetii* infection via metagenomic next-generation sequencing: a case report

**DOI:** 10.1186/s12879-022-07309-2

**Published:** 2022-04-13

**Authors:** Jingjia Zhang, Yan Hao, Zhi Wang, Qiwen Yang

**Affiliations:** 1 Department of Clinical Laboratory, Peking Union Medical College Hospital, Peking Union Medical College, Chinese Academy of Medical Sciences, Beijing, China; 2 Department of Cosmetic and Plastic Surgery, Peking Union Medical College Hospital, Beijing, China

**Keywords:** *Coxiella burnetii*, mNGS, Q fever, Case report

## Abstract

**Background:**

*Coxiella burnetii*, the etiologic agent of Q fever, is mainly responsible for endocardite. But there are only a few cases of *Coxiella burnetii*-caused wound infection have been published, because the pathogen is very difficult to isolate using conventional culture methods.

**Case presentations:**

A 76-year-old man, underwent endovascular repair of ruptured left iliac aneurysm plus abdominal aortic aneurysm under general anesthesia in 2018. Left iliac fossa mass resection was performed in 2020. After operation, the wound in the left iliac fossa was repeatedly ruptured and not healing. We used the wound tissue to perform the Metagenomics next-generation sequencing (mNGS), *Coxiella burnetii* was detected. Sanger sequencing and serologic verification of *Coxiella burnetii* all showed positive results.

**Conclusions:**

This study proved that mNGS was an effective method to detect clinically unexplained infections, and showed the ability of pathogen identification with high sensitivity and accuracy.

**Supplementary Information:**

The online version contains supplementary material available at 10.1186/s12879-022-07309-2.

## Background

Q fever is a zoonotic infection caused by the intracellular surpathogen *Coxiella burnetii* [1]. *Coxiella burnetii* is a classic strict intracellular, Gram-negative bacterium, which can usually be isolated from domestic animals including cattle, sheep, goats, cats, and dogs [2]. The bacterium can survive for a long period in dust. Humans are usually infected by aerosols, but sometimes may be infected through the digestive tract transmission, percutaneous exposure, transfusion or sexual intercourse [3]. Symptoms of Q fever can be acute and/or chronic. Q fever can be asymptomatic in around 60% of cases. In severe cases, it can lead to death [4, 5].

In vitro culture of *Coxiella burnetii* is very difficult, the culture medium formulation is complex and the culture time is very long [6]. Its culture is more demanding for the laboratory environment, and it failure to grow in standard blood culture. In the early stages of acute Q fever illness, a laboratory diagnosis can be made on the basis of serologic and Polymerase chain reaction (PCR) results. For a definitive diagnosis in the early stages of acute Q fever illness, serologic testing in combination with Polymerase chain reaction (PCR) is recommended [7]. But given that a significant proportion of infections are asymptomatic, it is difficult for clinicians to make a definitive request for a diagnosis of *Coxiella burnetii*. Metagenomic analysis has been used to diagnose the causes of many diseases with negative blood cultures or negative cultures of infected sites [8–10]. mNGS can realize the screening of most infectious pathogens and it has been used in the diagnosis of other pathogens such as *Orientia tsutsugamushi* [11], *Parvimonas micra* [12] etc. In this case, *Coxiella burnetii* was determined to be the causative pathogen by mNGS (non targeted metagenomics was performed not 16S targeted metagenomics) for Q fever. The result guided the clinical medication and treatment effectively.

## Case presentations

A 76-year-old male, underwent endovascular repair of ruptured left iliac aneurysm plus abdominal aortic aneurysm under general anesthesia in the first affiliated Hospital of Suzhou University in February, 2018. One month later, the patient developed left lower limb arterial embolism and osteofascial compartment syndrome. Then left lower extremity arterial thrombectomy plus stent implantation, debridement and suture of left lower limb osteofascial compartment syndrome were performed. A pulsatile mass with a diameter of about 6 cm in the left groin was found in March, 2020, with pain. Left iliac fossa mass resection and abdominal aortic angiography were performed on March 30, 2020. No aneurysm was found during the operation, and pathology suggested Fibrous cyst wall-like tissue, which had nothing to do with the femoral artery. Postoperative pathology showed fibrous cystic wall-like tissue, local granulomatous inflammation with massive necrosis. After operation, the wound in the left iliac fossa repeatedly ruptured and did not heal. Debridement was performed in the local hospital on May 29, 2020, and yellowish turbid fluid exudation was seen during the operation. It was considered that the wound may have deep infection and was not sutured. During the course of the disease, the patient had no discomfort such as fever, fear of cold, etc. He was admitted in the Plastic surgery, Peking Union Medical College Hospital on June 23, 2020.

**Fig. 1:**
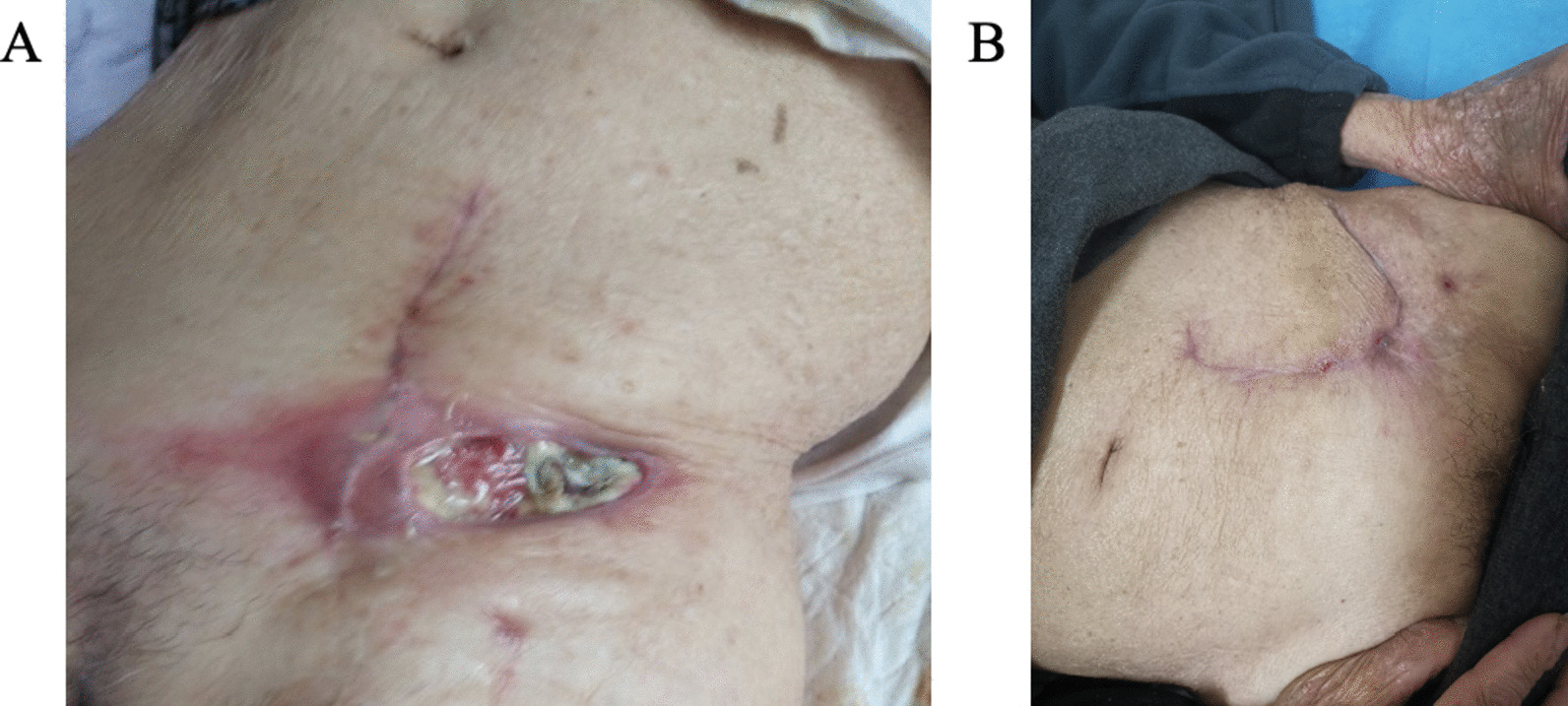
**A** and **B** showed the differences before and after treatment of the wound

During the period of hospitalization in our hospital, surgical examination revealed an 8 × 5 cm dehiscence wound in the left groin, necrotic pus moss in the internal muscles (Fig. 1A), no clear sinus tract was found in the deep tissue. Biological results indicated a raised C-reactive protein (CRP) (84.91 mg/L). We gave the patient bedside debridement and continuous negative pressure wound therapy using a vacuum sealing drainage (VSD) equipment. Debridement of the left iliac fossa, skin graft of thick-edged and skin removal of the right thigh were performed under general anesthesia on July 1, 2020, and the wound exudates were collected for bacteriological culture. VSD was routinely removed after skin graft surgery on July 7, 2020. The results of microbiological identification of wound exudates on July 6, 2020 were: *Escherichia coli* (ESBL +) and *Enterococcus faecalis*. The wound dressing was changed every day, and then the bacteria, fungi, tuberculosis/non-tuberculosis mycobacterium culture of the wound secretion were all negative. Due to the slow healing of the patient's wound, the VSD was placed again providing continuous negative pressure suction on July 22, 2020. The left iliac fossa wound debridement and local skin flap transfer repair were performed under local anesthesia on August 5, 2020.

**Fig. 2:**
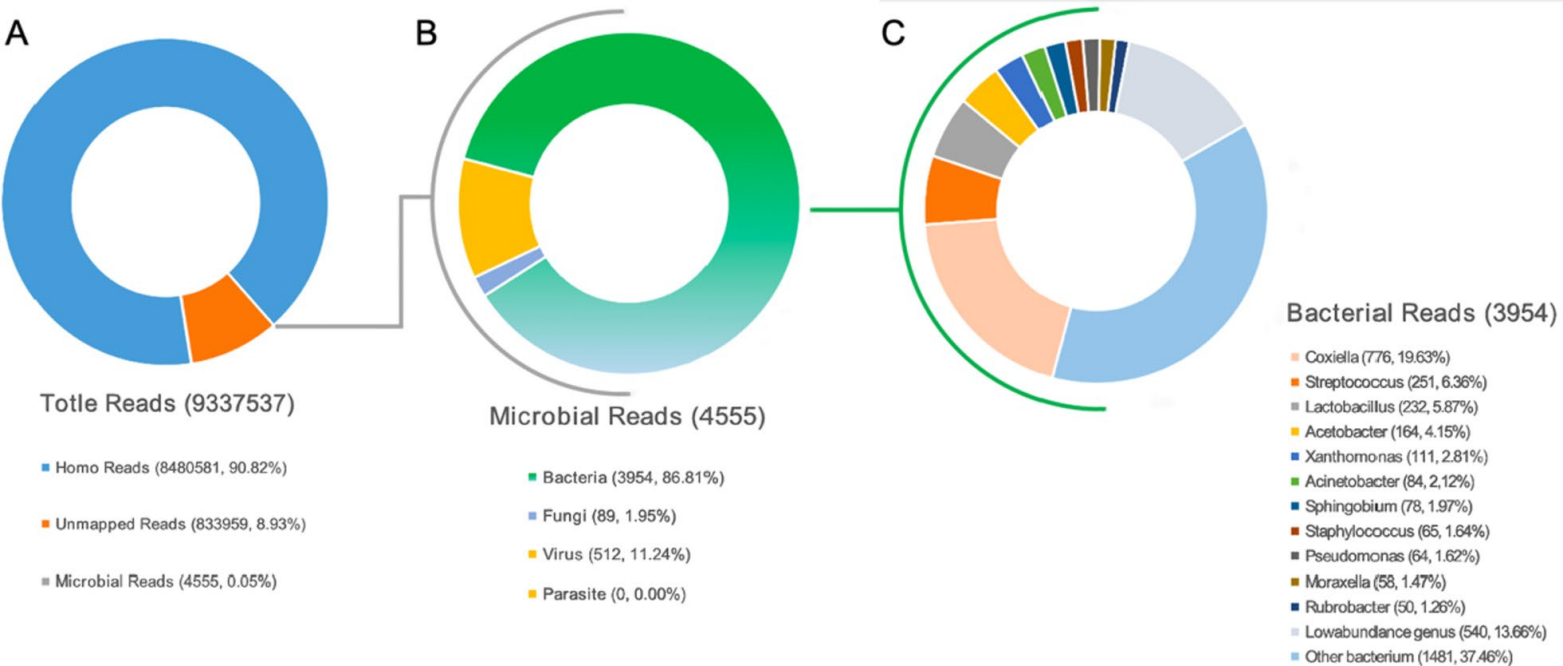
Taxonomic classification of reads by bioinformatic analysis of sequenced data from wound tissue

**Fig. 3:**
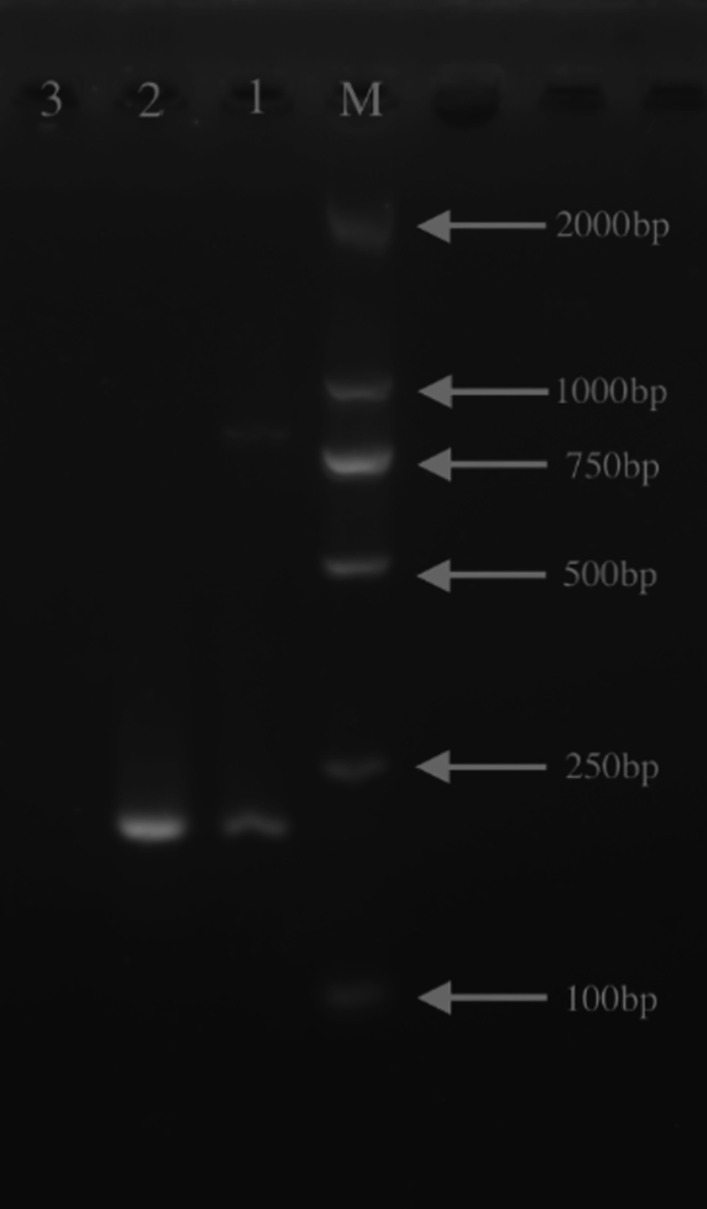
Agarose gel electrophoresis of PCR products derived from the *Coxiella burnetii IS1111a* gene. Gel electrophoresis of end-point PCR products (202 bp). M: DL2000 DNA marker (TAKARA); Line1: DNA sample from plasma; Line2: DNA sample from wound tissue; Line3: negative control

Considering that it might be a rare infection, we used the wound tissue and plasma to performed the metagenomics next generation sequencing (mNGS). For tissue sample, host DNA depletion procedure and conventional non-depletion procedure were both carried out before DNA library preparation. Briefly, tissue sample was sheared into small pieces by sterile scissors, then suspended in 400 μL saline. 200 μL sample was used for host DNA depletion according to manufacturer’s instructions, followed by ultrasonic disruption and DNA library preparation. DNA extraction and library preparation were still conducted on the NGSmaster™ library preparation system (Matridx, Hangzhou, China). The rest of tissue suspension without host DNA-depletion proceed with ultrasonic disruption and DNA library preparation as well. The concentration of prepared NGS libraries were determined by KAPA qPCR according to manufacturer’s instructions (Roche, Basel, Switzerland) followed by pooling. Single-end shotgun sequencing was carried out on NextSeq 500 platform using NextSeq Reagent Kit v2 (75-cycles) (Illumina, San Diego, CA, USA). Two days later, 776 sequence readings of *Coxiella burnetii* were detected in the wound tissue. The analysis of mNGS yielded a total of 9,337,537 single-end reads. The number of microbial reads was 4555, the number of bacterial reads was 3954 (86.81% of microbial reads). *Coxiella burnetii* had the highest relative abundance of 19.63% (Fig. 2). However, the result of the plasma was negative. PCR of the wound tissue and plasma was further tested for verification (using Q fever-*IS1111a* primers [13]), and both results were positive (Fig. 3). The straps from electrophoresis of PCR products were reclaimed by glue reclaim technology, and then the reclaimed straps were used to do sanger sequencing, both of the nucleic acid sequences belonged to *Coxiella burnetii.* Meanwhile, we carried out serological test by Immunofluorescence assay, and the result was Q fever IgM + (Additional file 1: Fig. S1).

**Fig. 4:**
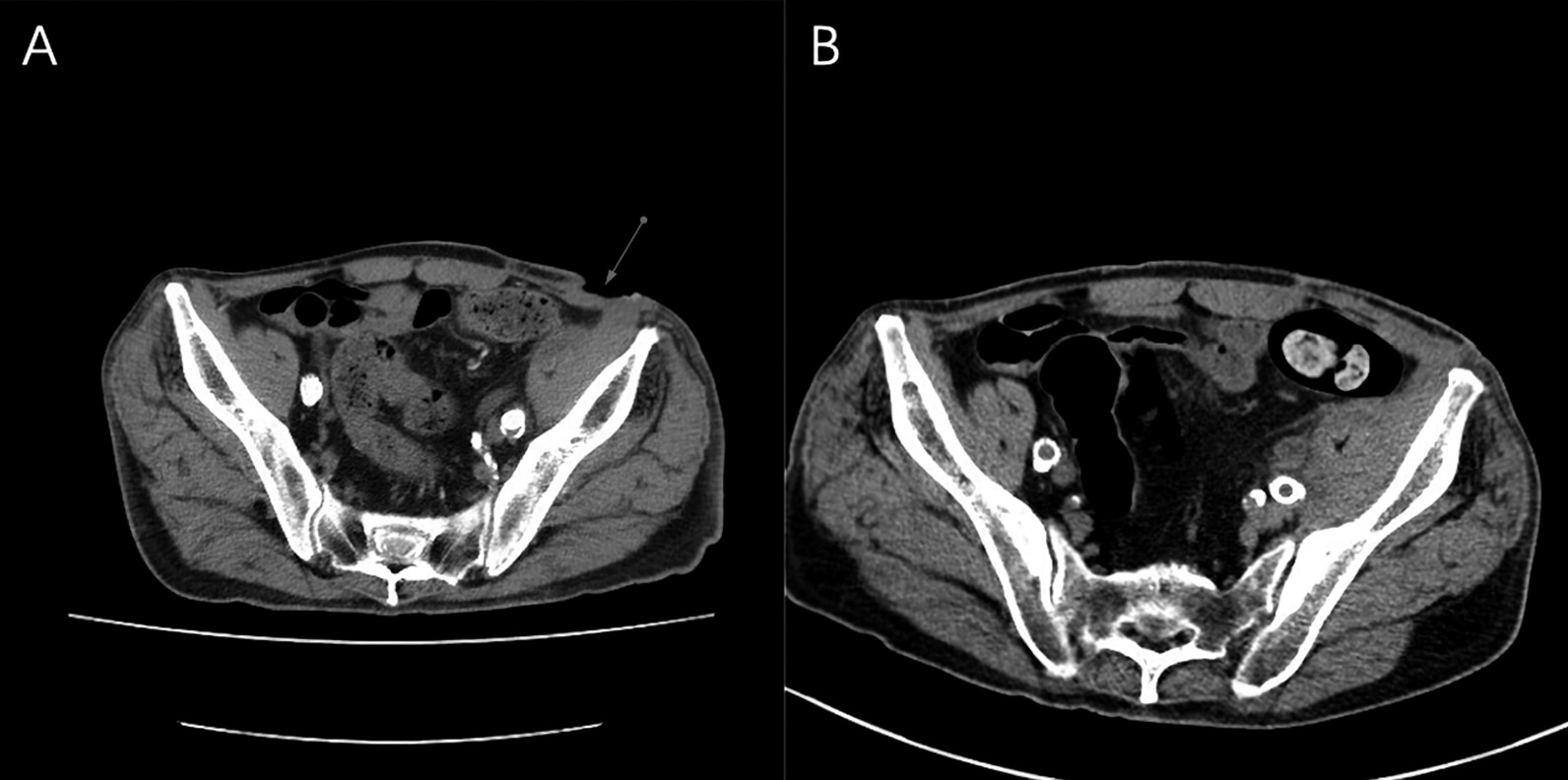
**A** and **B** were the abdominal CT before and after treatment, respectively. In figure **A**, the red arrow pointed to the obvious skin discontinuity, and the same part in figure **B** had healed

According to the consultation of infection internal medicine experts, it was considered that there was a great possibility of chronic Q-fever infection, and its aneurysm and cellulitis may be related to *Coxiella burnetii* infection. Doxycycline 0.1 g Q12h and hydroxychloroquine 0.2 g tid were recommended for treatment for about 1.5 years. The incision healed (Figs. 1B, 4) during hospitalization and the stitches were removed successfully on August 14, 2020. He was discharged from hospital on August 16, and started oral doxycycline and hydroxychloroquine for anti-Q fever.

Unfortunately, the patient died two months after discharge from our hospital due to massive hemorrhage in another hospital. The cause of death may be ruptured aneurysm or could be a relapse of the abscess with blood vessels invasion and subsequent rupture the blood vessels. According to previous reports [14, 15], the aneurysm with *Coxiella burnetii* infection tend to have a poor prognosis.

## Disscusion and conclusion

*Coxiella burnetii* is the agent of Q fever, first described in Australia in 1937 [7, 16]. Since this first description, knowledge about this pathogen and its associated infections were increased dramatically [16]. The diagnosis of infection requires the occurrence of an organic lesion (identified as the infectious focus) and the evidence of a microbial infection (as proven by serologic findings, polymerase chain reaction analysis, culture, and/or immunohistochemical analysis using anti-*Coxiella burnetii* antibodies) [16–18].

Prior to this time, *Coxiella burnetii* was detected in other types of infection by Sanger sequencing and serological tests. Chenouard et al. [19] reported a case of a Prosthetic Joint Infection associated with *Coxiella burnetii*. The diagnosis was performed by 16S rDNA sequencing. Godinho et al. [20] reported a kidney transplant recipient infected with *Coxiella burnetii*, diagnosed by Q fever serological test. In this case, *Coxiella burnetii* was first detected on the wound by mNGS in China and then validated by Sanger sequencing and serologic test. The traditional culture method is not suitable for the detection of *Coxiella burnetii* since there is no commercial medium for cultivating *Coxiella burnetii* on the present market, and the culture cycle is very long, about more than a week. Sanger sequencing and serologic test are targeted detection methods, which need to be designed according to the suspected pathogen. mNGS is an groundbreaking technology that can sequence most genes at once in a specimen, and then match them with existing microbial gene bank to detect pathogens. Compared with traditional PCR diagnostic techniques, mNGS undoubtedly have advantages, such as the ability to detect bacteria, fungi, parasites and viruses simultaneously in a single test, and could obtain a large number of microorganisms to distinguish pathogens. mNGS is particularly suitable for identifying rare pathogens that cannot be cultured.

Futhermore, *Coxiella burnetii* was positive in wound tissue and negative in plasma detected by mNGS. But *Coxiella burnetii* was positive in both specimens by PCR method. The bands from electrophoresis of PCR products showed that the wound tissue was brighter than the plasma (Fig. 3). Obviously, the bacterial load in the wound tissue was higher than the plasma. Therefore, the detection sensitivity of mNGS may not as good as PCR.

In summary, it was difficult to diagnose coxiellosis timely owing to the limitations in conventional diagnostic methods. mNGS was a new diagnostic technology which could accurately and fast identify *Coxiella burnetiid*, it would be a very useful tool to find the aetiology of unidentified infections.

## Supplementary Information

**Additional file 1: Figure S1.** Fluorescence image of serum Q fever antibody detected by immunofluorescence method, green fluorescence is displayed after the antibody binds to the Q fever antigen.

## Data Availability

All the data in this study are included in the published articles.

## Abbreviations

mNGS: Metagenomics next-generation sequencing
PCR: Polymerase chain reaction
CRP: C-reactive protein
VSD: Vacuum sealing drainage
ESBL: Extended-Spectrum β-Lactamases
DNA: Deoxyribonucleic acid
qPCR: Quantitative Real-time PCR
