# Supplementary material for: Diagnosis of Coxiella burnetii infection via metagenomic next-generation sequencing: a case report
Source: BMC Infect Dis. 2022 Apr 13;22:373. doi: 10.1186/s12879-022-07309-2 (PMC9008969; doi:10.1186/s12879-022-07309-2)
Supplement: Supplementary file 1 — Additional file 1: Figure S1. Fluorescence image of serum Q fever antibody detected by immunofluorescence method, green fluorescence is displayed after the antibody binds to the Q fever antigen. [file 12879_2022_7309_MOESM1_ESM.docx]

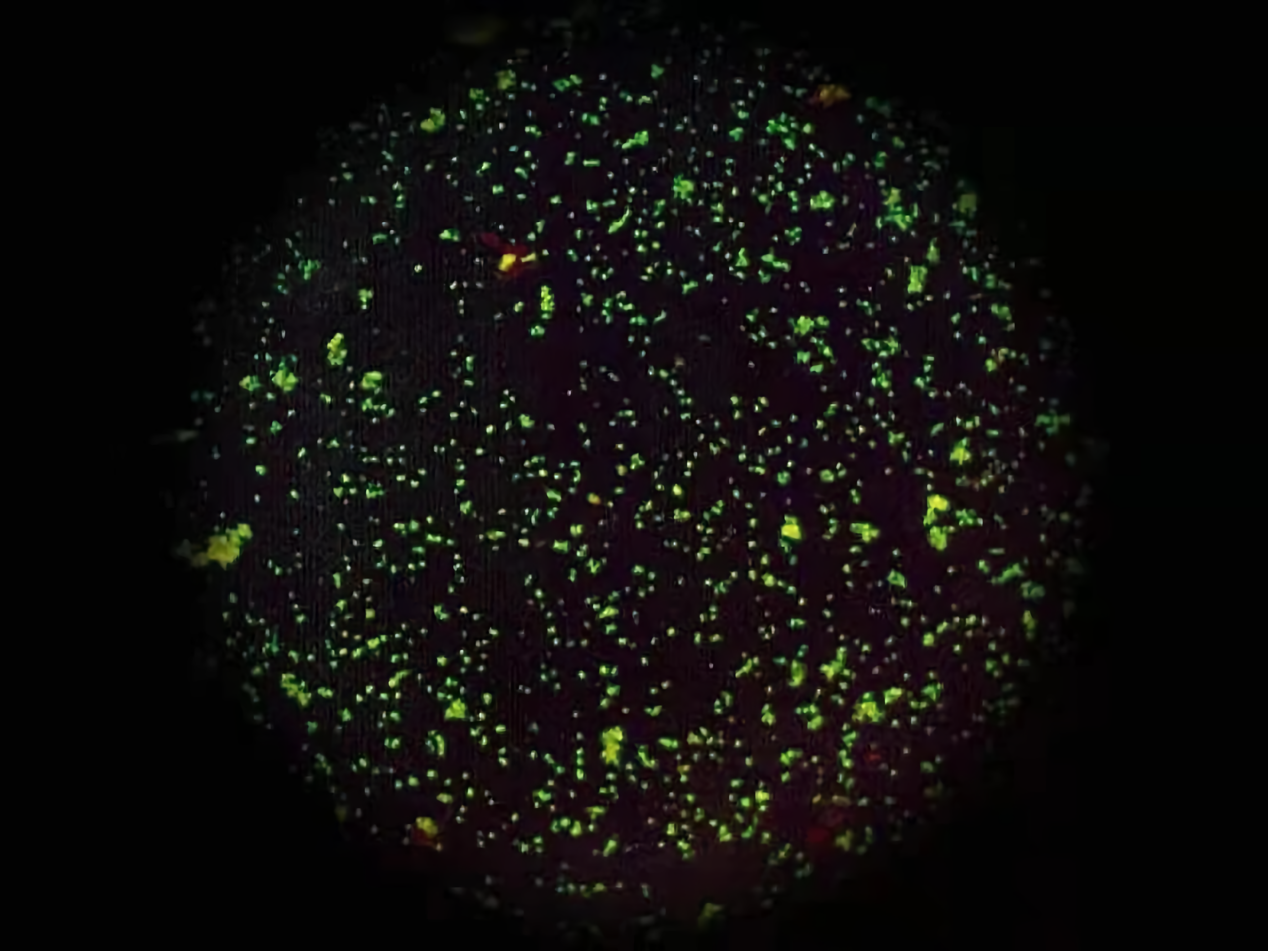


Figure S1. Fluorescence image of serum Q fever antibody detected by immunofluorescence method，green fluorescence is displayed after the antibody binds to the Q fever antigen.
